# Determinants for swine mycoplasmal pneumonia reproduction under experimental conditions: A systematic review and recursive partitioning analysis

**DOI:** 10.1371/journal.pone.0181194

**Published:** 2017-07-25

**Authors:** Beatriz Garcia-Morante, Joaquim Segalés, Emmanuel Serrano, Marina Sibila

**Affiliations:** 1 IRTA, Centre de Recerca en Sanitat Animal (CReSA, IRTA-UAB), Campus de la Universitat Autònoma de Barcelona, Bellaterra, Spain; 2 UAB, Centre de Recerca en Sanitat Animal (CReSA, IRTA-UAB), Campus de la Universitat Autònoma de Barcelona, Bellaterra, Spain; 3 Departament de Sanitat i Anatomia Animals, Facultat de Veterinària, Campus de la Universitat Autònoma de Barcelona, Bellaterra, Spain; 4 Servei d’Ecopatologia de Fauna Salvatge, Departament de Medicina i Cirurgia Animals, Universitat Autònoma de Barcelona, Bellaterra, Spain; 5 Departamento de Biologia and Centro de Estudos do Ambiente e do Mar, Universidade de Aveiro, Aveiro, Portugal; The University of Melbourne, AUSTRALIA

## Abstract

One of the main *Mycoplasma hyopneumoniae* (*M*. *hyopneumoniae*) swine experimental model objectives is to reproduce mycoplasmal pneumonia (MP). Unfortunately, experimental validated protocols to maximize the chance to successfully achieve lung lesions induced by *M*. *hyopneumoniae* are not available at the moment. Thus, the objective of this work was to identify those factors that might have a major influence on the effective development of MP, measured as macroscopic lung lesions, under experimental conditions. Data from 85 studies describing *M*. *hyopneumoniae* inoculation experiments were compiled by means of a systematic review and analyzed thereafter. Several variables were considered in the analyses such as the number of pigs in the experiment, serological status against *M*. *hyopneumoniae*, source of the animals, age at inoculation, type of inoculum, strain of *M*. *hyopneumoniae*, route, dose and times of inoculation, study duration and co-infection with other swine pathogens. Descriptive statistics were used to depict *M*. *hyopneumoniae* experimental model main characteristics whereas a recursive partitioning approach, using regression trees, assessed the importance of the abovementioned experimental variables as MP triggering factors. A strong link between the time period between challenge and necropsies and lung lesion severity was observed. Results indicated that the most important factors to explain the observed lung lesion score variability were: (1) study duration, (2) *M*. *hyopneumoniae* strain, (3) age at inoculation, (4) co-infection with other swine pathogens and (5) animal source. All other studied variables were not relevant to explain the variability on *M*. *hyopneumoniae* lung lesions. The results provided in the present work may serve as a basis for debate in the search for a universally accepted *M*. *hyopneumoniae* challenge model.

## Introduction

*Mycoplasma hyopneumoniae* (*M*. *hyopneumoniae*) has been recognized as the etiological agent of enzootic pneumonia (EP) and a relevant infectious agent of the porcine respiratory disease complex (PRDC). Both are chronic respiratory diseases affecting mainly grow-finishing pigs, causing major economic losses in the pig industry worldwide[1]. Mycoplasmal pneumonia (MP) is grossly characterized by purple to grey areas of pulmonary consolidation, mainly found bilaterally in the apical, cardiac, intermediate and the anterior parts of the diaphragmatic lobes [2]. Such lung lesions can be experimentally induced in pigs inoculated with *M*. *hyopneumoniae* [3].

*M*. *hyopneumoniae* experimental swine models have been primarily used to study the pathogenesis of the infection as well as to evaluate the efficacy of antibiotics or vaccines [3]. However, variation in the outcome of such experimental models (in terms of number of animals affected by MP and its severity) is a common drawback. In fact, in this scenario, clinical course and pattern of *M*. *hyopneumoniae* infection have been described to be dependent on several factors such as co-infection with other pathogens [4–6], study duration [7, 8], differences in virulence between *M*. *hyopneumoniae* strains [9, 10], inoculum type [11], dose [12] or inoculation route [13]. The abovementioned factors together with other conditions highly differ between published experiments, becoming difficult to elucidate specific conclusions regarding their influence on MP development and severity. As a result, a reference-validated model for the experimental MP reproduction in swine is still unavailable. In consequence, the present study sought to identify determinants for the successful reproduction of MP under experimental settings. To reach this goal, a systematic review approach was used for the comprehensive assessment of *M*. *hyopneumoniae* experimental model characteristics as well as for identification of relevant studies. Subsequently, recursive partitioning was applied to the compiled data to evaluate the influence of experimental conditions on lung lesion outcomes.

## Materials and methods

### Data source and literature review

The protocol for the current systematic review is in accordance with the Preferred Reporting Items of Systematic Reviews and Meta-analysis (PRISMA) guidelines (see S1 PRISMA Checklist) [22]. The search term “*Mycoplasma hyopneumoniae*” was used in Medline^®^/PubMed^®^ database (https://www.ncbi.nlm.nih.gov/pubmed) for peer-reviewed articles describing *M*. *hyopneumoniae* swine experimental models from January 1990 to May 2016. Those relevant citations contained links to full-text content from PubMed Central (PMC), National Center for Biotechnology Information (NCBI) Bookshelf and different publisher web sites. Only articles written in English were considered. In addition, those pertinent studies cited in the reference list of the abovementioned articles were also systematically reviewed. The term “study” was used to define a published *M*. *hyopneumoniae* experimental inoculation work and “experimental unit” to define a particular set of experimental conditions applied in one or more pigs within a study. While in some studies a unique set of conditions was applied, others included more than one experimental unit.

### Study inclusion criteria

Eligible studies were those providing: 1) information in regards of MP prevalence and/or macroscopic score (considering also those with no success for MP achievement); and 2) at least one experimental unit including *M*. *hyopneumoniae*-inoculated pigs (with or without co-infection with other swine pathogens). Therefore, those experimental units in which pigs were mock inoculated, immune stimulated (through vaccination or adjuvantation) or contact-exposed to *M*. *hyopneumoniae* were not considered. On the contrary, experimental units within a study meeting the two abovementioned inclusion criteria were systematically introduced into a database (Excel 2013 software, Microsoft Office®).

### Data extraction and adequacy

Several variables were annotated from each experimental unit accomplishing the aforesaid inclusion criteria (Table 1). For the inoculation route (InRoute) variable, the term intratracheal (IT) referred to those methods placing the inoculum directly into the trachea, including both endotracheal and transtracheal systems. In regards to *M*. *hyopneumoniae* strain (Strain) variable, the term “pool” was applied in those experimental units in which more than one strain were used simultaneously or subsequently to inoculate the animals. In addition, for the additional challenge with other swine pathogens (CoIn), it was considered either when *M*. *hyopneumoniae* was co-inoculated before, at the same time or after another bacterial or viral microorganism. Lastly, a major setback of the systematic review process was the fact that different lung lesion scoring systems were used along the different compiled studies. In consequence, recently reported formulae of equivalence were applied to homogenize mean lung lesion scores between experimental units [14]. Thus, all scores were converted into the European Pharmacopoeia score (Ph. Eur., monograph number 04/2013:2448), which expresses the weight percentage of affected lung tissue by MP.

**Table 1:** Name, description, abbreviation and classification of the variables annotated within experimental units and included in the statistical analyses. Levels within categorical variables are also indicated.

Variable	Description	Abbreviation	Classification	Variable Levels	Abbreviation
Sample size	Number of pigs included in an experimental unit	SamSize	Continuous	—	—
Age at inoculation	Weeks of age of the animals when inoculated	InAge	Continuous	—	—
Inoculation dose	Amount of *M*. *hyopneumoniae* (expressed in color changing units; CCU) given to each animal	Dose	Continuous	—	—
Days of inoculation	Number of days in which the inoculum is given	InDays	Continuous	—	—
Necropsy	Post-inoculation week in which animals were necropsied and lung lesions evaluated	Nweek	Continuous	—	—
Lung lesion prevalence	Proportion of affected lungs within an experimental unit	Llprop	Continuous	—	—
Lung lesion score	Mean lung lesion score (Ph. Eur.) in each experimental unit expressed as weight percentage of affected tissue	Llscore	Continuous	—	—
Seronegativity	Seronegative for *M*. *hyopneumoniae* before inoculation	SerNeg	Categorical	Yes	—
				No	—
Source of animals	Source and health/immunological status of the animals	Asource	Categorical	Conventional	C
				Caesarean-derived, colostrum-deprived	CDCD
				Specific-pathogen free	SPF
Route of inoculation	Route used for the inoculum administration to the animals	InRoute	Categorical	Intratracheal	IT
				Intranasal	IN
				Aerosol	AE
*M*. *hyopneumoniae* strain	*M*. *hyopneumoniae* strain used for challenge	Strain	Categorical	—	—
Type of inoculum	Type of challenge material	InType	Categorical	Lung homogenate	LH
				Culture	CT
				Lung homogenate and pure culture mixed	LH+CT
Co-infection	Additional challenge with other swine pathogens	CoIn	Categorical	Yes	—
				No	—

### Data analyses

#### Univariate statistics

Descriptive statistics were used to analyze variables and summarize their main characteristics (Excel 2013 software, Microsoft Office®). Distribution, central tendency and dispersion parameters were calculated for continuous variables whereas frequency distribution bar charts were created for categorical variables.

#### Multivariate statistics

The contribution of the previously mentioned variables to the experimental induction of MP was assessed through a recursive partitioning analysis via decision tree models construction. Thus, tree-based models were grown and pruned by using the *rpart* 4.1–10 package [15] in the R software version 3.3.2 [16]. The pruning procedure reduces the size of the original tree, simplifying and facilitating its interpretation and avoiding overfitting data [17, 18]. In the present work, decision trees were pruned according to a complexity parameter (CP), defined as the cost (in terms of relative error reduction) of adding another variable to the model. Hence, models were started with a low CP value (CP = 0.001) and afterwards, the most parsimonious trees were obtained by using the CP obtained from the model with the lower prediction error after 10 cross validations. In addition, 12 experimental units were the minimum number of observations needed to generate a node. Lastly, an overall measure of variable importance (VIP) and the percentage of the observed MP variability, retained in the final and most parsimonious regression tree models, were calculated. A generalized additive model [19], which describes the non-linear relationships via nonparametric smoothing functions, was applied to further investigate associations between variables. This model was constructed using the R software 3.3.2 version [16] and the *mgcv* 1.8–12 package [20].

## Results

### Included studies

**Fig 1:**
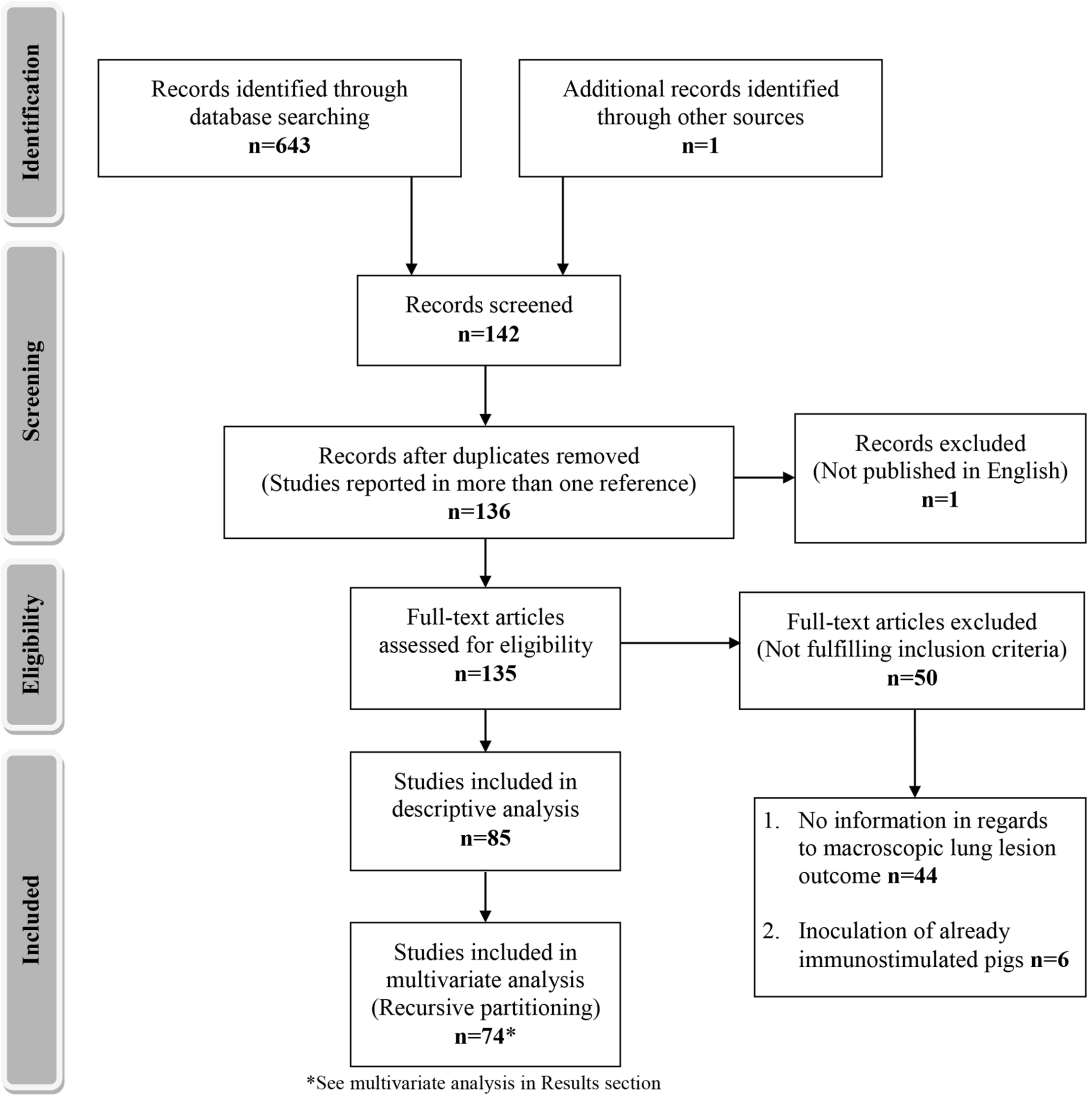
Flowchart depicting the flow of information (records identified, included and excluded) through the different phases of the systematic revision. PRISMA guidelines were followed [22].

Study selection flow diagram is shown in Fig 1. Electronic search in Medline^®^/PubMed^®^ database produced an initial list of 643 references until 1990 (last entry in May 2016). An additional article written in Chinese was identified in other sources [21]. Examination of titles, abstracts and reference lists in the retrieved articles led to a preliminary list of 142 potentially relevant studies (*M*. *hyopneumoniae* experimental inoculations reported), which, after removal of duplicities, were reduced to 136. After examination of full text, 85 studies, including a total of 261 experimental units, complied with the inclusion criteria and therefore, they were included in the analyses.

### Descriptive analysis

**Fig 2:**
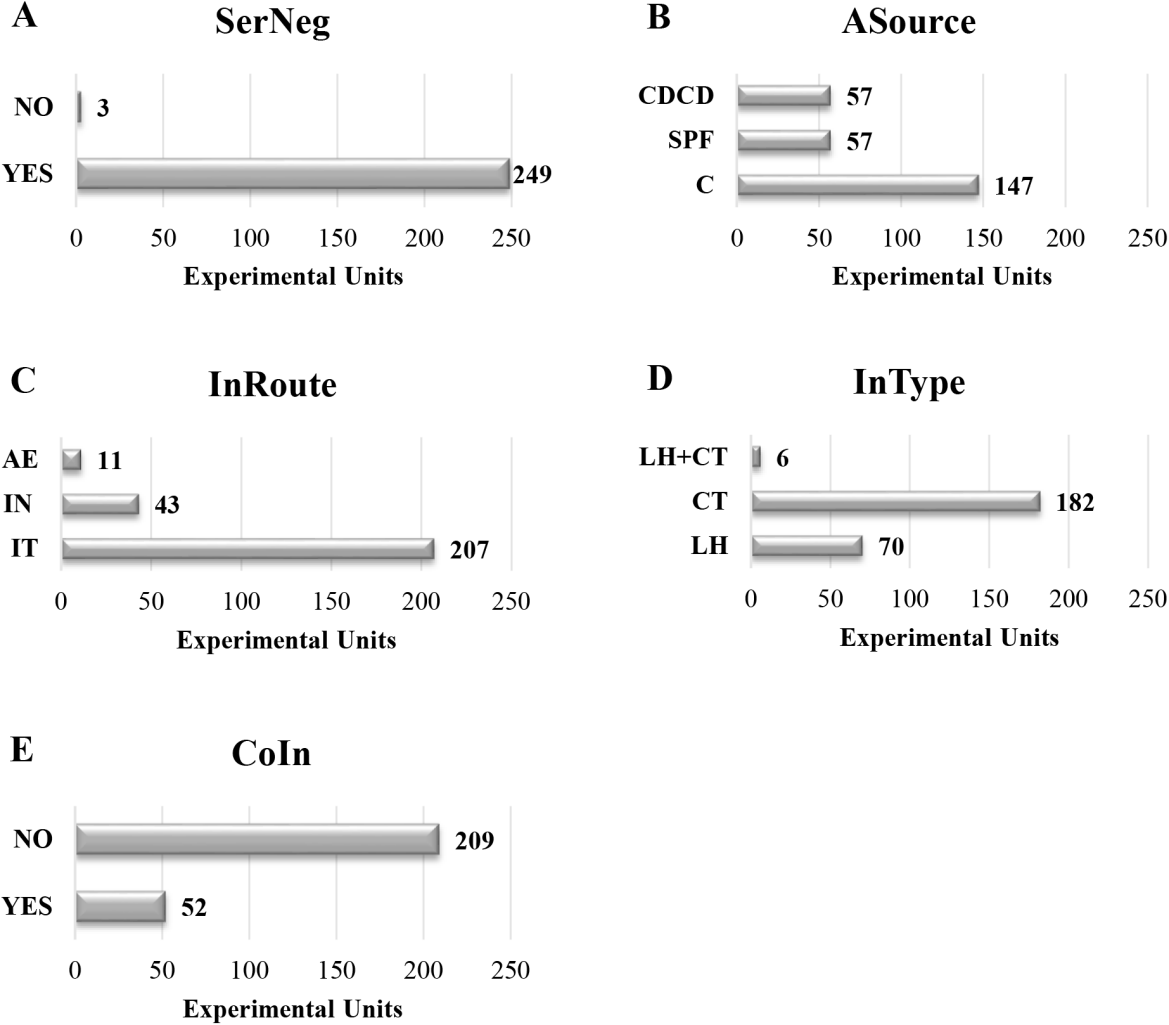
Frequency distribution bar charts of SerNeg (A), ASource (B), InRoute (C), InType (D) and CoIn (E) categorical variables.

**Fig 3:**
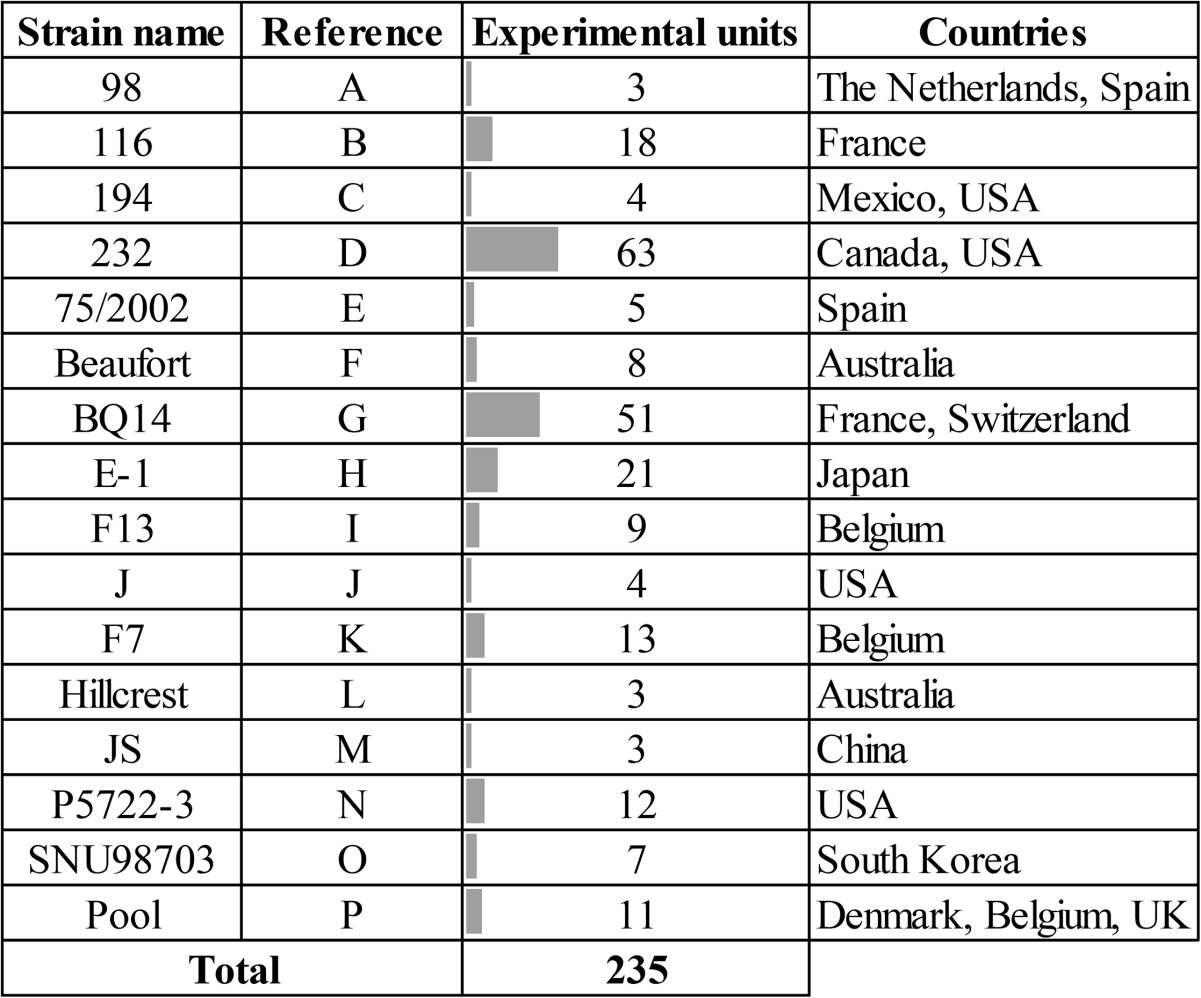
Frequency distribution bar chart along experimental units of those well-defined *M*. *hyopneumoniae* strains, the given alphanumerical reference and the countries where their use has been reported.

Since different variables were specified in each experimental unit, the number of experimental units considered for the analyses differed between variables. Main descriptive statistics calculated for continuous variables and the number of experimental units considered for each variable is summarized in Table 2, whereas frequency distribution bar charts for categorical variables are shown in Fig 2. Only those strains appearing in at least three experimental units not coming from the same study were considered for analyses. Fig 3 displays the frequency distribution bar chart of considered *M*. *hyopneumoniae* strains together with an alphanumerical reference and the countries where they were used.

**Table 2:** Descriptive statistics calculated for continuous variables within each experimental unit and the number of experimental units considered for each variable.

			Distribution	Central tendency			Dispersion
	No. of experimental units	Units	Range	Mean	Median	Mode	Standard deviation
**SamSize**	261	No. of pigs	1–116	7.66	5	1	10.10
**InAge**	258	Week	1–17	6.04	6	6	3.61
**Dose**	234	CCU/pig	2.19–1.50 x 10^15^	3.21 x 10^13^	7 x 10^8^	10^7^	2.17 x 10^14^
**InDays**	261	Day	1–6	1.56	1	1	0.91
**Nweek**	252	Week	1–52	6.29	4	4	6.85
**Llprop**	160	Percentage	0–100	71.96	100	100	39.60
**Llscore**	227	Percentage	0–51.82	9.83	8.01	0	9.10

The mean sample size within experimental units was approximately of 8 inoculated pigs. Nonetheless, experimental units including only one pig were the most prevalent ones. The latter is probably explained because of the impact of a unique study with numerous experimental units of 1 pig each [8]. Conventional pigs were used in more than the half of the experimental units and free-*M*. *hyopneumoniae* antibody animals were principally chosen. In regards of the inoculation procedure, pigs were often challenged at 6 weeks of age through the IT inoculation route in one time. Concerning the inoculum, *M*. *hyopneumoniae* broth culture (CT) was applied in the majority of cases. The dosage given per pig was greatly variable, reaching a maximum of 1.50 x 10^15^ CCU [23]. Such a large dose displaced the mean to a high value, but the median dose per animal was 7 x 10^8^ CCU, being 10^7^CCU/pig the most prevalent inoculum dose. Nearly one quarter of the experimental units used co-infection with other viral and bacterial swine respiratory pathogens. In decreasing order of importance, the pathogens co-inoculated were *Porcine reproductive and respiratory syndrome virus*, *Swine influenza virus*, *Porcine circovirus type 2* and bacterial pathogens such as *Actinobacillus pleuropneumoniae* or *Pasteurella multocida*. The mean study duration was found to be about 6 weeks, although in most of the cases the animals were necropsied after 4 weeks post-inoculation (wpi). As outcomes, the mean percentage of lungs affected by MP within an experimental unit was 72% and the mean lung lesion score (Ph. Eur.) was around 10% of lung weight damaged. Importantly, from the 261 included experimental units, 30 (11.5%) did not manage to reproduce MP. The use of a given strain was found to be closely related with the laboratory or research group. *M*. *hyopneumoniae* 232 was the most largely used strain, mainly by the United States research groups. In Europe, a higher variability is seen, but French *M*. *hyopneumoniae* BQ14 and 116 strains were the most prevalent through experimental units from this continent. Notably, the use of a pool of *M*. *hyopneumoniae* strains, meaning simultaneous or subsequent inoculations of at least two different strains, has been reported in experimental inoculation models only in Europe. In Asia, the Japanese E-1 strain was found to be the most commonly used one. Lastly, two different strains were handled in Australia, named Beaufort and Hillcrest.

### Multivariate analysis

Since the Llscore variable was more commonly specified within experimental units (n = 227) than the Llprop one (n = 160), the former was chosen as response variable in the multivariate analysis (74 studies considered). The explanatory variables were: InAge, Asource, InRoute, Strain, InType, Dose, InDays, CoIn and Nweek. Giving the fact that only 3 experimental units from 2 studies used *M*. *hyopneumoniae* seropositive pigs [24, 25], SerNeg variable was not considered in recursive partitioning.

**Fig 4:**
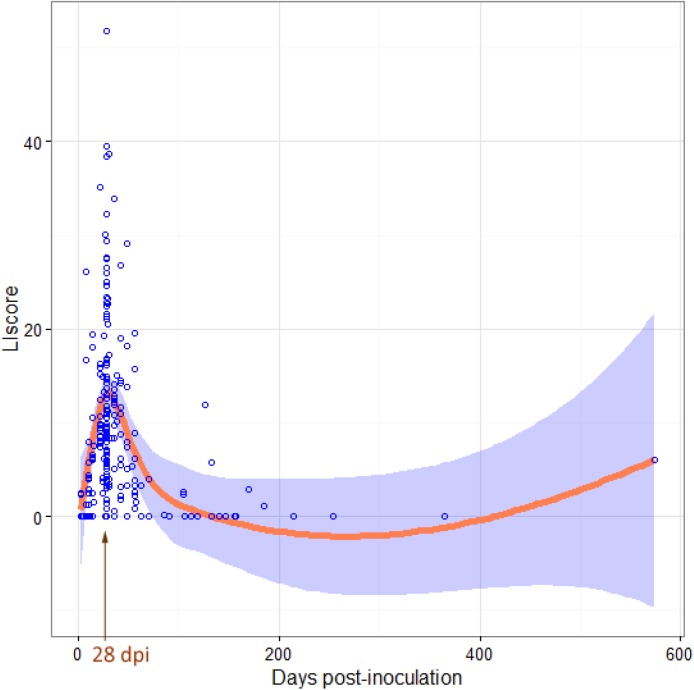
Plot representing the distribution of values for the Llscore variable depending on the day post-inoculation (dpi) at which animals were necropsied. Blue dots represent Llscore values (n = 227). The arrow indicates that Llscore peaked when necropsies were performed at 28 dpi. The orange line and the blue shadow symbolize the predictions and their range of the additive model (GAM) at 95% confidence interval, respectively.

**Fig 5:**
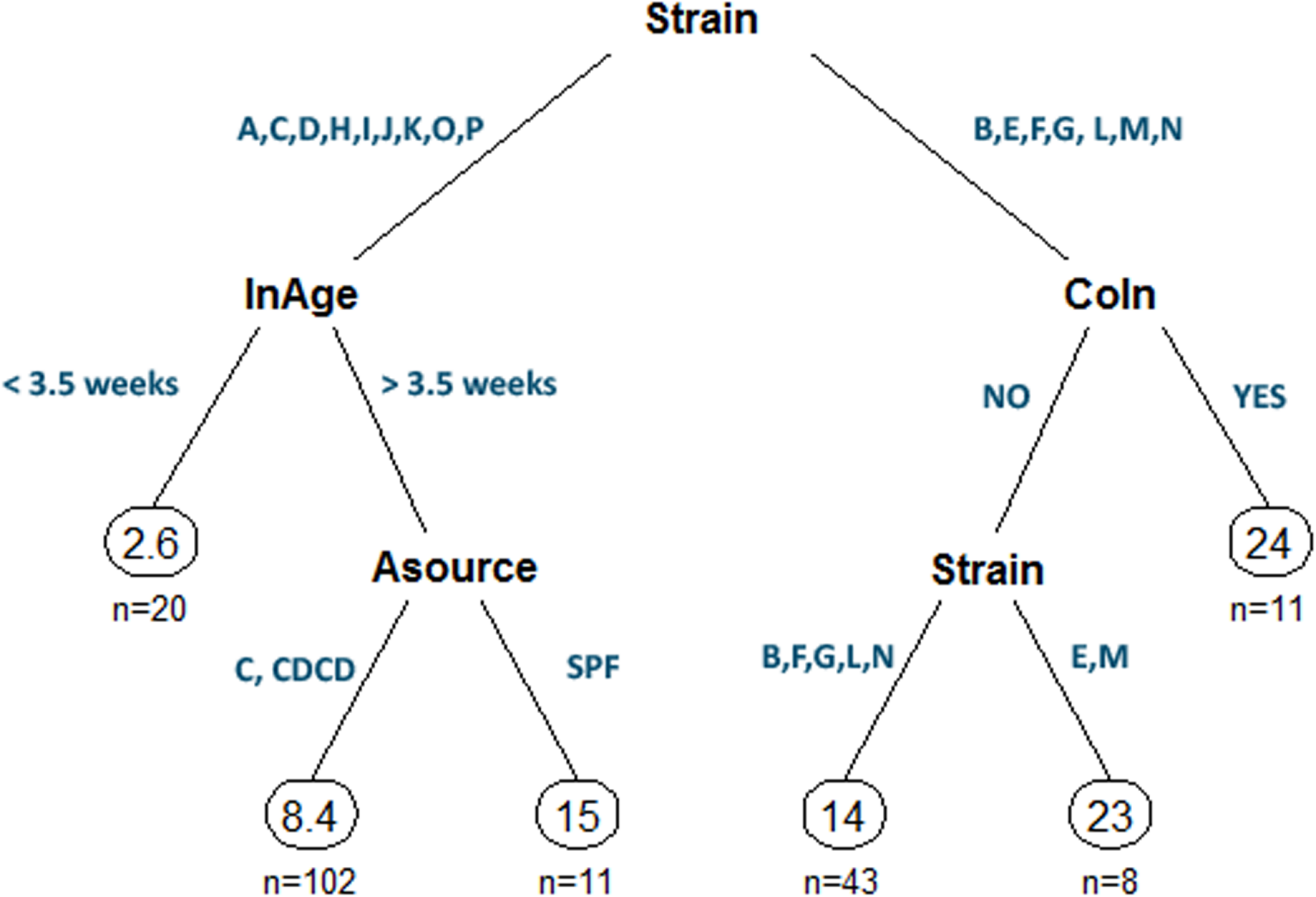
Final regression tree resulting from experimental units with a maximum duration of 8 weeks post-inoculation. The numbers into the circles represent the mean value of Llscore variable in “n” experimental units. Blue values aside the lines represent the different categories of the corresponding variables.

In a first obtained tree-based model, in which all explanatory variables were fitted to get the full picture of the model, Nweek was the most important variable by far as shown by an *ad hoc* ANOVA test (F_9, 215_ = 6.36, *p*-value = 5.485e-08, R^2^ = 21%). Using GAM, the strong connection between Nweek and Llscore variables was reinforced (F = 10.71, edf = 5.95, *p*-value = 3.34e-11, R^2^ = 30%). Indeed, Llscore peaked when necropsies were performed at 4 wpi, dropping later at 8 wpi (Fig 4). Thus, the first obtained decision tree established that successful protocols were those assessing MP below 8wpi; specifically, the top ten Llscore were obtained between 3 and 7 wpi. Consequently, and in order to enable the identification of other putative influencing factors on the Llscore variability, another tree-based model was obtained from the adjustment of data from experimental units with a maximum duration of 8 wpi. As a result, the final decision tree explained 33% of the observed Llscore variability (Fig 5) and, in decreasing order of importance, the factors to be accounted to successfully reproduce MP in experimental units of duration below8 wpi were the following: Strain (VIP = 67), InAge (VIP = 18), CoIn (VIP = 9) and finally, Asource (VIP = 5). Overall, *M*. *hyopneumoniae* strains 116, 75/2002, Beaufort, BQ14, Hillcrest, JS and P5722-3 resulted in higher Llscore values than all the other assessed strains. Importantly, some of the aforesaid strains lead to the highest Llscore in co-infected pigs (Llscore = 24%, n = 11).Nonetheless, sole inoculation with 75/2002 or JS strains resulted in similar Llscore values (Llscore = 23%, n = 8) to those reached in co-infection. All the remaining strains resulted in lower Llscore outcomes, particularly when the age of pigs at inoculation was lower than 3.5 weeks (Llscore = 2.6%, n = 20).When above 3.5 weeks of age, inoculated specific pathogen-free (SPF) pigs showed higher Llscore values (Llscore = 24%, n = 11) than their C or cesarean-derived, colostrum-deprived (CDCD) counterparts (Llscore = 8.4%, n = 102).All the other analyzed variables, namely InRoute, InDays, InType and Dose,werenot found relevant to explain Llscore.

## Discussion

Numerous attempts to induce MP at experimental level have been reported and, as evidenced by the present results, variation in both the number of animals affected by MP as well as in severity of such lung lesions do occur. The latter is accompanied by wide differences between *M*. *hyopneumoniae* experimental models, though highlighting the existing inconstancy between protocols. Works studying how a unique variable may influence MP under experimental settings have been already made [7, 8, 10–13]. Contrarily, observation and analysis of more than one experimental variable at a time has not been assessed. In consequence, a combination of experimental factors that would increase the chance to successfully achieve MP is not available and the uncertainty around this problem is still far of being mitigated. Therefore, the present work investigated which experimental conditions might be accounted for reproduction of MP, determined by the macroscopic lung lesion score, and proposes the initial bases to build are producible and effective *M*. *hyopneumoniae* challenge model pursuing this goal.

In order to accomplish the abovementioned objective, data from a series of studies were summarized through a systematic review and analyzed by means of recursive partitioning. To authors’ knowledge, this is the first time that such statistical method has been applied to evaluate the performance of experimental infections in pigs. Along the same line, Tomás *et al*. (2008) identified factors with a relevant influence on the expression of clinical postweaning multisystemic wasting syndrome (PMWS) under experimental conditions, albeit different statistical techniques were applied [26]. Recursive partitioning is particularly well suited for exploring complex relationships, where there is often little knowledge nor predictions regarding which and how variables are related [17]. In the present work, recursive partitioning was used to create a decision tree that defined an easy to visualize linear combination of continuous and categorical explanatory variables (InAge, Asource, InRoute, Strain, InType, InDays, Dose, CoIn and Nweek) to predict a continuous response variable (Llscore). Moreover, recursive partitioning provides a better ability to handle missing values in both explanatory and response variables than other regression techniques [18]. Missing data was perhaps one of the most important limitations of the present systematic review, being common within experimental units of different studies. For instance, the pig genetics could not be considered because in most works the breed used was not specified.

In the first fitted regression tree containing all explanatory variables, the most determinant factor associated to the presence of lung lesions in experimental trials was found to be the study duration (Nweek). Henceforth, and to further examine such relationship, a complementary analysis was performed by means of GAM. In this way, GAM analysis revealed that lung lesion scores peaked when the Llscore was assessed at 4 wpi, being congruent with those studies indicating that macroscopic lung lesions reach their maximum level around that moment [7, 8]. Hence, the present results support this lapse of time as the standard for the assessment of lung scores. However, it is worth mentioning that the infection model of 4 wpi might not be suitable for all types of experimental conditions [13, 27]. In fact, results indicated that the time period that should not be exceeded to evaluate lung pathology is 8 wpi. Accordingly, those experiments quantifying lung lesions over 8 wpi aimed principally to assess *M*. *hyopneumoniae* transmission and persistence instead of lung pathology [28–30].Taking into account all the above, final tree models were applied to data from experimental units of less duration than 8 wpi, and the most relevant factors to be accounted for inducing lung lesions suggestive of MP appeared to be the *M*. *hyopneumoniae* strain (Strain) followed by the age of animals at inoculation (InAge), co-infection with other swine pathogens (CoIn) and, finally, the source of the animals (Asource).

Differences in virulence between *M*. *hyopneumoniae* strains have been described [9, 10]. Despite antigenic and genetic differences between isolates have been demonstrated [31, 32], it is still not known whether such differences correlate with dissimilarities in virulence [10, 33, 34]. By recursive partitioning, the *M*. *hyopneumoniae* strains assessed were split into two groups, one leading to higher lung lesion score outcomes than the other. Thus, the obtained results support the already reported high virulence of some *M*. *hyopneumoniae* strains such as Hillcrest [23] or even the lack of pathogenicity of J strain [35, 36], from which some current *M*. *hyopneumoniae* commercial vaccines are based [37]. On the contrary, the American 232 and the Belgian F7, recognized as high virulent strains [9, 38], were found less appropriate for severe MP reproduction than other *M*. *hyopneumoniae* strains. As a distinctive condition, simultaneous or subsequent inoculations with more than one strain did not result in more severe lung lesions. Although some studies have suggested an enhanced effect of multiple infections with different *M*. *hyopneumoniae* strains [27, 34, 39], our analyses support findings reported by Charlebois *et al*. (2014), in which no link was found between lungs harboring more than one strain and lung lesion severity. Notwithstanding, given that data from single *M*. *hyopneumoniae* strain inoculations predominate over data from multiple strain co-inoculations, the obtained results should be corroborated. Additionally, differences between *M*. *hyopneumoniae* strains might be explained, among others, by the number of *in vitro* passages, which has been discussed to result in pathogenicity differences [36]. Unfortunately, this factor could not be addressed because it was not explicitly mentioned in an extensive number of publications.

Immunopathological events are considered to play an imperative role in the pathogenesis of *M*. *hyopneumoniae* infection [1, 40]. Consequently, the higher potential of success obtained for inoculations in pigs older than 3.5 weeks of age could be explained by the fact that some mechanisms from both the innate and acquired immunity are less developed at birth and mature with age [1]. Indeed, under conditions of a previous study [41], no differences were detected in susceptibility of pigs to *M*. *hyopneumoniae* between 3 and 12 weeks of age. Furthermore, in almost all the analyzed studies, inoculated pigs were tested to be seronegative against *M*. *hyopneumoniae*, excluding conferred protection of younger pigs by passively acquired maternal antibodies. While *M*. *hyopneumoniae* is considered a primary respiratory pathogen, thus, able to induce disease by itself, the use of co-infections have been also proven effective to induce *M*. *hyopneumoniae*-like lung lesions in several experiments. However, whether co-infection with other swine pathogens influences the severity of *M*. *hyopneumoniae* infection (macroscopic lung lesions) is still under debate. Whereas some studies found no potentiation of pneumonia by other microorganisms [4, 6, 25, 42], others demonstrated that pneumonia in co-inoculated pigs was more severe than in pigs inoculated only with *M*. *hyopneumoniae* [5, 43, 44]. Despite such discrepancies, results of the recursive partitioning analysis indicate that co-infection models might have the potential for reproducing pneumonia than the inoculation of *M*. *hyopneumoniae* alone. Regrettably, the lack of more experimental units using co-infection models hindered the evaluation of the co-inoculated pathogens separately, being all of them pooled and evaluated together. The latter might be taken into account since the lung lesion outcome may vary depending on the co-inoculated microorganism. Lastly, the use of SPF pigs resulted to be an important feature to effectively reproduce MP. Unfortunately, criteria applied to define SPF pigs by the authors reporting their use were unknown, becoming difficult to precisely explain the importance of such factor on lung lesion outcome. Nevertheless, and independently of the source, pigs used in *M*. *hyopneumoniae* experimental models were, in general terms, *M*. *hyopneumoniae*-free by means of serology and/or polymerase chain reaction (PCR) techniques. Hence, pigs naive to *M*. *hyopneumoniae* are probably required to increase the chance to achieve severe MP.

All the other evaluated variables (InRoute, InDays, InType and Dose) were found not relevant to explain MP variability. Contrarily, higher percentage of animals affected by MP as well as mean lung lesion score have been reported in pigs inoculated endotracheally in comparison with pigs inoculated intranasally or by aerosolization [13]. Inoculation with lung homogenate (LH) taken from infected pigs might has the potential to introduce adventitious agents as well as aggravate the inflammatory response due to the administration of foreign antigens [11]. However and in accordance to the obtained results, no significant differences were observed among animals inoculated with LH and CT prepared from the same lung homogenate in terms of lung lesions achieved [45]. Furthermore, an inoculum dose-dependent response has been reported in *M*. *hyopneumoniae* challenge studies, in which very high doses are necessary to achieve clinical disease [12]. In agreement, descriptive statistics revealed an overall high dose (CCU) used per pig.

Importantly, care must be taken in drawing conclusions from the obtained results. The statistical approach performed in the present study was probably hampered by the broad differences in the applied conditions between experimental units, which make it difficult to assess the effect of a particular condition in the lung lesion outcome. Moreover, inherent limitations of the included publications and the systematic review process must be also taken into account. In this respect, the trend towards publishing only positive results (e.g. experiments in which MP was successfully achieved) and the exclusion of those studies that did not fulfil all the systematic review inclusion criteria (e.g. experiments in which MP was induced but no information in regards of lung lesion score was provided), probably lead to biases that could have influenced the obtained results. Another important reason for bias is linked to the high prevalence of some particular conditions within a variable, such as the lung lesion assessment at 4 wpi as striking example. Thus, the importance of such widely extended conditions within *M*. *hyopneumoniae* experimental inoculation protocols could be overestimated in the statistical analysis.

## Conclusion

The present study broadens the current understanding in regards to *M*. *hyopneumoniae* experimental swine models and constitutes the first insight into conditions needed to induce lung lesions suggestive of MP under experimental settings. Thus, obtained results might serve as a basis for debate in the search for a *M*. *hyopneumoniae* experimental model seeking to induce severe MP. While the *M*. *hyopneumoniae* strain used may depend basically on the strain availability, other easily modifiable conditions might be taken into account in experimental models. Therefore, the highest likelihood to achieve severe MP would require lung lesion assessment within a period below 8wpi and include inoculation of SPF (*M*. *hyopneumoniae* free) pigs older than3.5 weeks of age and, preferably, in co-infection with another swine respiratory pathogen as a triggering factor.

## Supporting information

S1 PRISMA Checklist Systematic review checklist. Protocol followed in accordance with the PRISMA guidelines.
(DOC)

S1 Data Final database used for the statistical analyses. (XLSX)
